# Editorial: Crosstalk: skin cells and immune cells in inflammatory skin diseases, volume 2

**DOI:** 10.3389/fimmu.2025.1739229

**Published:** 2025-11-19

**Authors:** Kazuhiko Yamamura, Akira Shiraishi, Emi Sato, Samuel Williams

**Affiliations:** 1 Department of Dermatology, Kyushu University, Fukuoka, Japan; 2 Department of Pediatrics, Kyushu University, Fukuoka, Japan; 3 Department of Dermatology, Fukuoka University, Fukuoka, Japan; 4 Department of Internal Medicine, University of California, San Diego, San Diego, United States

**Keywords:** skin, immune cells, neuron, inflammation, crosstalk

The skin, the body’s largest organ, acts as a dynamic interface between the external environment and internal homeostasis. Beyond its well-known roles in barrier protection and sensory perception, the skin functions as an active immune organ that coordinates closely with systemic immune networks. This Research Topic, *“Crosstalk – Skin Cells and Immune Cells in Inflammatory Skin Diseases, Volume 2,”* expands upon the first volume to further explore the multilayered relationships among epithelial, immune, and neural networks in the skin. This volume includes eight original and review articles that bridge basic discoveries and clinical implications in inflammatory dermatology.

## Neuro–immune crosstalk in atopic dermatitis

In the field of atopic dermatitis (AD), two studies advanced our understanding of neuro–immune interactions. Sato et al. compared the short-term (3-month) clinical efficacy of a JAK1 inhibitor and an anti–IL-13 antibody using real-world data. Although there was no significant difference in EASI-75 response or adverse events within this period, the JAK1 inhibitor demonstrated stronger antipruritic effects. Distinct biomarkers correlated with each treatment: eosinophil reduction in the JAK1i group and TARC reduction in the IL-13Ab group. These findings provide valuable insights for biomarker-driven personalized therapy in the era of molecularly targeted treatments. Ishikawa et al. revealed that oncostatin M (OSM) promotes sensory nerve elongation and cutaneous hypersensitization, proposing the OSM pathway as a novel therapeutic target for chronic itch. Together, these studies deepen our understanding of the neuro-immune crosstalk that drives AD pathogenesis.

## Immune dysregulation and systemic involvement in psoriasis

In psoriasis research, Yao et al. provided an integrative review of the epidermal immune microenvironment (EIME), discussing its disruption from genetic, epigenetic, microbiome, and neuroimmunological perspectives. Their synthesis highlights how the IL-23/IL-17 axis sustains chronic inflammation and contributes to systemic comorbidities. Šabović et al. demonstrated that, even during treatment, psoriasis patients may retain a procoagulant state due to impaired fibrinolysis, underscoring the link between cutaneous inflammation and systemic vascular risk. Morino et al. reported that the SULT2B1–cholesterol sulfate–DOCK2 pathway plays a pivotal role in maintaining skin homeostasis by suppressing excessive immune activation. This study introduces a new epithelial–immune regulatory axis relevant to psoriasis.

## Broader perspectives on inflammatory and immune-mediated diseases

Saito-Sasaki and Sawada reviewed the lifestyle and environmental factors that affect hidradenitis suppurativa (HS), emphasizing that diet, obesity, and sleep patterns influence immune balance and disease activity. He et al. identified a pathogenic IL-17-producing T-cell subset in HLA-B27-positive juvenile idiopathic arthritis (JIA). The authors linked these cells to destructive synovial inflammation and proposed that IL-17 signaling could be a potential therapeutic target. Finally, Uno et al. described the immunomodulatory function of hyaluronan tetrasaccharide (HA4) in the skin. HA4 suppresses inflammatory macrophage polarization while maintaining fibroblast-mediated collagen production, suggesting potential applications in skin regeneration and antifibrotic therapy.

## Conclusion

Collectively, these studies redefine the skin as a “boundary organ” governed by multilayered crosstalk among epithelial, immune, neural, metabolic, and environmental networks. Volume 2 bridges the gap between fundamental and clinical dermatology and provides perspectives that may guide future advances in drug discovery, personalized medicine, and inflammation control.

